# Survival of patients with chronic heart failure in the community: a systematic review and meta-analysis protocol

**DOI:** 10.1186/s13643-018-0810-x

**Published:** 2018-10-03

**Authors:** Nicholas R. Jones, Andrea K. Roalfe, Ibiye Adoki, F. D. Richard Hobbs, Clare J. Taylor

**Affiliations:** 1 Nuffield Department of Primary Care Health Sciences, University of Oxford, Radcliffe Primary Care Building, Radcliffe Observatory Quarter, Woodstock Rd, Oxford, OX2 6GG England; 2 Foundation Training Programme, Oxford University Hospitals NHS Foundation Trust, John Radcliffe Hospital, Headley Way, Headington, Oxford, OX3 9DU England

**Keywords:** Heart failure, Prognosis, Primary care, Systematic review, Meta-analysis

## Abstract

**Background:**

Heart failure (HF) is a common condition affecting more than 10% of those over 70 years of age. Reliable estimates of survival following a diagnosis of HF are important to guide management and facilitate advanced care planning. Most existing research has focused on survival rates for patients admitted to hospital with acute HF. However, the majority of patients with HF are diagnosed in the outpatient setting and can have periods of sustained symptom stability in the chronic phase of their illness. There has not been a systematic review of the literature to determine the prognosis of patients with chronic HF in the community.

**Methods:**

We will undertake a comprehensive search of the following databases: CINAHL, Database of Abstracts of Reviews of Effects, Embase, MEDLINE and the Clinical Trials Register (clinicaltrials.gov). Two reviewers will independently complete screening, data extraction and quality appraisal with the option of input from a third reviewer to arbitrate. We will include data from observational or database studies conducted in either community or outpatient settings. Studies of acute HF or specific subgroups of patients will be excluded. There is no restriction by geographical setting, publication language or study date. We will complete QUIPS and GRADE assessments to systematically appraise the quality of evidence within and between studies. Where possible, we will seek to pool results to conduct a meta-analysis and undertake relevant subgroup analysis including by study setting, participant age and study decade. The primary outcome will be survival time from diagnosis. The secondary outcomes will be HF-related hospital admissions, symptom burden and measures of morbidity.

**Discussion:**

This systematic review will provide up to date evidence on the current survival rates and prognostic indicators for patients with chronic HF. We will put this into historical perspective, comparing outcomes across time to help understand the impact of advances in evidence-based treatment on average survival. This information is important in facilitating informed decision-making for patients and health professionals as well as highlighting areas to focus resources and improve public health planning.

**Systematic review registration:**

PROSPERO 2017 CRD42017075680

**Electronic supplementary material:**

The online version of this article (10.1186/s13643-018-0810-x) contains supplementary material, which is available to authorized users.

## Background

Heart failure (HF) has been defined as a global pandemic affecting between 1 and 2% of the adult population [1] and over 10% of people aged 70 years or older [2]. Incidence is expected to continue to rise due to demographic changes and increasing prevalence of HF risk factors, such as hypertension and ischaemic heart disease. HF carries a significant morbidity and mortality equivalent to many forms of cancer, and quality of life is worse than in most other chronic diseases [3]. The economic burden is also significant: In the United Kingdom (UK), HF accounts for around 2% of the National Health Service (NHS) budget [4], second only to stroke in terms of expense. The high prevalence, costs and poor outcomes make HF an important public health problem.

HF has traditionally been divided into ‘acute’ and ‘chronic’ HF. ‘Acute’ HF occurs when there is an episode of deterioration in symptoms, which may include the time of initial diagnosis [5]. The European Society of Cardiology (ESC) define ‘chronic’ HF where patients have had HF ‘for some time’ and ‘stable’ after 1 month or more of stable symptoms [5]. Patients will often spend long periods of time in this chronic, stable phase of their illness, especially in the early stages following diagnosis and initial treatment. In the UK and many European countries, patients with HF will have the majority of their treatment based on the community [6]. The typical pattern of disease is for the periods of relative stability to be interrupted by increasingly frequent acute decompensations, which require a step up in care [7]. Acute and chronic HF may therefore be seen as a spectrum of disease. Frequent hospital admissions and episodes of acute HF are themselves a poor prognostic marker and sign of progressive disease.

Most previous studies of HF prognosis have focused on ‘acute’ HF and recruited patients who have been admitted to hospital following a recent deterioration in symptoms. These results are not directly generalisable to patients in the stable, chronic phase of their condition. One year survival in acute HF is between 55 and 65% [8, 9], compared to 80 to 90% in chronic HF [10, 11]. Understanding the disease trajectory, mean survival times and prognostic markers specific to chronic HF is important. The chronic HF phase should be an opportunity to treat comorbidities, optimise medication, promote lifestyle changes and discuss prognosis with patients to enable them to undertake meaningful advanced care planning. Accurate prognostic information is essential to inform patients, their families and healthcare professionals during this decision-making process [7]. Prognostic information is also important when considering the allocation of resources in healthcare planning.

Treatment for HF has changed over time and evidence-based medications are now available that reduce hospital admissions and mortality for patients with some forms of HF. HF is categorised by the left ventricular ejection fraction (LVEF). A patient with symptoms of HF and LVEF < 40% is defined as having HF with reduced ejection fraction (HFrEF), in contrast to symptoms with a LVEF ≥ 50%, which is defined as HF with preserved ejection fraction (HFpEF). A LVEF between 40 and 50% is defined in the recent ESC HF guidelines as HF with mid-range ejection fraction (HFmrEF). Beta-blockers (BB) [12], angiotensin-converting-enzyme inhibitors (ACEI) [13] and mineralocorticoid receptor antagonists (MRA) [14] have all been shown to reduce morbidity and mortality for patients with HFrEF. No treatment has yet been shown to improve survival rates in HFpEF. Audit data from hospitals shows significant reductions in mortality rates for patients with acute HF over the past two decades since the introduction of these treatments [15]. There was an expectation amongst clinicians and researchers that survival in chronic HF would similarly improve but our recent work suggests it may be relatively unchanged [11]. Clarifying the temporal trends for prognosis in chronic HF is essential for understanding the real-world impact of evidence-based treatment in the community setting.

Analysis of the patients included across the studies can help identify which factors are associated with prognosis in chronic HF. There are numerous recognised markers of poor prognosis, including reduced ejection fraction, ventricular arrhythmias, renal impairment and low functional capacity. However, data about the relative importance of these and their applicability to patients with chronic HF is lacking. A better understanding of prognostic markers could help focus treatment strategies and enable more accurate survival data to be applied to specific patient subgroups.

Previous studies of HF prognosis have been conducted internationally across heterogeneous patient cohorts. We will attempt to locate, assess and summarise all existing, relevant studies relating to survival for patients with chronic or stable HF, treated either in primary care or the secondary care outpatient setting. This information can help to identify areas for potential improvement in current HF management as well as areas for future research. We anticipate the findings being relevant to patient groups, policymakers and healthcare providers in both primary and secondary care. This paper outlines the rationale and describes the methodology for the planned systematic review.

## Methods

### Objectives

This systematic review aims to determine the prognosis for adult patients with a diagnosis of HF who are in the ‘stable’ or ‘chronic phase’ as per ESC 2016 guidelines [5]. The primary outcome is years of survival following a diagnosis of chronic HF. Secondary outcomes will include hospital admissions, symptom burden including morbidity measures and factors associated with an increased risk of death including age, left ventricular systolic dysfunction, treatment and comorbidities.

### Protocol and registration

The protocol has been developed in accordance with the Cochrane Collaboration guidance [16] and conforms to the PRISMA-P (Preferred Reporting Items for Systematic Reviews and Meta-Analysis Protocols) recommendations [17]. A completed PRISMA-P checklist is included in Additional file 1. The protocol was submitted for registration on the International Prospective Register of Systematic Reviews (PROSPERO) website on 12 October 2017, ahead of any data extraction. PROSPERO registration CRD42017075680. The protocol is available from:

http://www.crd.york.ac.uk/PROSPERO/display_record.php?ID=CRD42017075680

### Eligibility criteria

#### Population

We will include adult patients (aged 18 years or older), with a confirmed diagnosis of HF and in the ‘stable’ or ‘chronic’ phase of their illness, as defined by ESC guidelines. There will be no restrictions based on the geographical location of the study or the publication language. We will exclude patients recruited following an episode of acute HF or decompensation, patients who have been recruited specifically on the basis of another condition (e.g. patients receiving dialysis), or specific sub-groups of HF including those with a likely reversible cause e.g. toxins or tachycardiomyopathy.

### Intervention

We seek to understand prognosis in the context of usual care and as such will exclude service evaluations or interventional studies, where the primary outcome is the efficacy of the trialled treatment. We will collect information on treatment where this is provided in the individual studies, but there is no restriction by the treatment patients are receiving during the study period.

### Outcome

The primary outcome is survival time. Where possible, we will include survival time from point of diagnosis, but where this information is not available, we will use survival time from the point of enrolment into the study as a surrogate. Studies where follow-up is under 1 year duration are excluded as we will be unable to extract information on longer-term prognosis.

### Exclusion criteria

We seek studies that are most likely to reflect real-world practice and provide data that are generalisable to community populations. On this basis, we will exclude interventional studies and focus on observational studies, database studies or studies that include both an interventional and observational phase. We will exclude case reports, case series, studies that focus on novel biomarkers or studies which have recruited selective sub-populations as these also lack generalisability beyond the study setting. We will exclude conference abstracts as these provide insufficient details of methodology to critically appraise. Studies which do not present original data, such as review articles, will be excluded but we will search their list of references for original research studies which do meet our inclusion criteria.

### Information sources and literature search

The following databases and trial registers will be included in the search: CINAHL, Database Abstracts of Reviews of Effects, Embase, MEDLINE and the Clinical Trials Register (clinicaltrials.gov). These databases will be searched from the earliest possible date on each database to September 2017. There is no limitation on the publication date as we hope to capture any change in prognosis over time and whether prognosis has improved since the introduction of evidence-based treatments. PubMed will not be searched given its overlap with MEDLINE. Web of Science will not be searched given its focus on technology studies. Cochrane Registry of Clinical Trials will not be searched given its focus on interventional studies. A search update will be planned within 3 months of completion of the systematic review.

The search strategy was developed with the support of the departmental librarian (NR) to try and ensure a comprehensive approach incorporating all key search and Medical Subject Headings (MeSH) indexation terms. The strategy was based around four search domains joined with the Boolean operator ‘AND’. These domains aimed to capture the condition, the outcome, the study type and the study setting. Search terms were based on previous systematic reviews, feedback from the librarian and the validated Scottish Intercollegiate Guidelines Network ‘Search filters for observational studies [18]. Following an initial validation search run for specificity and sensitivity, we included the additional terms of ‘general population’ and ‘epidemiology*’ to the study setting domain. The full MEDLINE search strategy can be found in the ‘Appendix’ section. References will be managed using the Endnote X7 (Thomson Reuters) software.

A hand search of the references of included studies will be carried out to ensure literature saturation. The results from the literature search will also be checked with local experts to ensure they reflect the core current evidence in the field. Where necessary, we will attempt to contact the authors where clarification is needed, e.g. over study design or incomplete results to maximise the reliability of the data collected and the chances of including all relevant material.

Grey literature will be excluded from the search strategy as the type of data that is being sought is almost exclusively published in peer-reviewed journals. Whilst a grey literature search would increase the sensitivity of the search, the additional resources required to do so would make this study unfeasible within its current limited resources.

### Study selection

Two reviewers (NJ, IA) will independently screen all titles and abstracts from the original search against the predefined eligibility criteria. We will obtain full-text versions of any papers that appear to meet the inclusion criteria or those where there is insufficient evidence in the title and abstract to make a decision (level 1 screening). The same two reviewers will then undertake a second round of independent full-text screening to decide on the final list of studies for inclusion (level 2 screening). Where there is a disagreement, the two will hold a discussion to come to a consensus on inclusion. If this is not possible, individual papers will be discussed and reviewed with a third reviewer (CT) to reach an agreement. A vote and majority decision can take place if necessary, if the three are unable to reach agreement. We will record the rationale for excluding any paper on the data extraction template where we have reviewed the full text. A PRISMA flow chart of this process will be included in the review.

### Data items and data collection process

We plan to extract the following information:

- Author information, publication date, publication language, geographical location, study setting and study dates.
- Study design, sample size and methodology.
- Definition of HF, echocardiography findings including left ventricular ejection fraction, participant average age, gender, comorbidities, treatment and mean duration of follow-up.
- Funding sources and conflict of interest declarations will also be recorded.
- Outcome data will include hospital admission rate, mortality rate, cause of death frequencies and summary statistics of any measure of morbidity, e.g. symptom score or measure of severity of HF across the study.
- Number of deaths and number of person-years at risk, by year of follow-up.
- Effect estimates for potential prognostic factors including age group, gender, ejection fraction categories, comorbidity (diabetes mellitus, myocardial infarction, angina, hypertension) and treatment (ACE inhibitors, beta blockers, diuretics, ARBs).

A data extraction template will be created in advance to allow for standardised data extraction and cross-checking of results in line with the data outlined in the study protocol. The data extraction template will be piloted on the first 10% of papers before being reviewed for completeness. Two reviewers (NJ, IA) will independently extract data from the included paper and then meet to compare and discuss any inconsistencies. Agreement on the independent screening and data extraction decisions will be quantified using kappa coefficients. A third reviewer (CT) will be available if required to reach a consensus opinion. No patient-identifiable information will be used. All information will be stored on encrypted computers and held according to Good Clinical Practice guidance.

### Methodological appraisal and risk of bias

The risk of bias and methodological quality of included studies will be assessed using the Quality in Prognosis Studies (QUIPS) tool [19], which is specifically designed for use in prognostic studies. The methodology of individual observational studies will also be assessed against the STROBE (Strengthening the Reporting of Observational Studies in Epidemiology) criteria [20]. Two separate reviewers (NJ, IA) will undertake the risk of bias assessment. The results will be included in the final report for readers’ information and we will also include a sensitivity analysis excluding low-quality papers to determine their impact on outcomes. A Grading of Recommendations Assessment, Development and Evaluation working group (GRADE) quality score will be undertaken to give an estimate of confidence in the cumulative outcomes [21]. GRADE is a standardised framework for making a systematic, transparent summary of evidence and presenting this in a manner that can help inform clinical practice. It is widely used in systematic reviews and recommended by the Cochrane Collaboration. The GRADE assessment considers eight key domains to give an estimate of overall confidence of the summary outcomes. Whilst observational studies are initially given a low GRADE score, they can be marked up if there is evidence of a dose-response gradient, a large effect size for the outcome or where residual confounding factors would be expected to increase the overall effect size. This means it is possible for some observational reviews to provide estimates with a high degree of confidence. GRADE recommends marking evidence down where there is evidence of risk of bias, imprecision, inconsistency, indirectness and publication bias. GRADE tables will be included in the final report to provide a clear and accessible synthesis of this process.

### Data synthesis

All data will be analysed using STATA version 14 software with specialist statistician input (AR) during the review stage. Summary tables will be included to present study information and key outcomes in a structured format. Meta-analysis will be performed, where possible, of percentage survival at various time points, e.g. 1 year, 5 years and 10 years, and results presented as forest plots. If sufficient data are available, a summary survival curve will be produced from survival probabilities and numbers of at-risk patients at time points across the studies. Heterogeneity will be assessed using the chi-squared and *I*^2^ statistics respectively and causes for heterogeneity sought and discussed, using sensitivity analysis where possible. We plan to undertake subgroup analysis by study setting, study decade, participant age, degree of left ventricular impairment and compliance with guideline-recommended medication and device use. Sensitivity analysis will be performed to exclude studies deemed of low methodological quality based on their QUIPs score. Forest plots will be included to test for publication bias. We plan to undertake the meta-analysis using random effects modelling since there will be some variability due to clinical and methodological differences between the studies. Meta-analysis of potential prognostic factors will be carried out including age at diagnosis, gender, left ventricular ejection fraction, comorbidities and treatment. If effect estimates such as log odds ratios or log hazard ratios and their corresponding standard errors are not directly available, we will estimate them using relevant data where possible. Adjusted and unadjusted effect estimates will be presented separately. Where studies use different cut-points and methods of measurement, we will perform a separate meta-analysis for each subset of studies that used the same measurement and cut-point. We will report any instances where it has not been possible to undertake a planned meta-analysis.

## Discussion

To our knowledge, there has yet to be a systematic review on this subject. Determining the current prognosis for patients with chronic HF is vital for patients, their families and healthcare providers to guide management and decisions regarding future care. The review will provide up-to-date information on key potential prognostic factors such as age, treatment, or left ventricular ejection fraction. Understanding modifiable and non-modifiable prognostic factors will help make this information relevant in guiding treatment strategies and could inform public health policy. Prognostic information may also enable healthcare providers to adapt treatment strategies in certain patient groups and provide more accurate and specific information on survival. Comparing changes in prognosis over time will give insight into the real-world impact of evidence-based interventions. If the prognosis is not improving over time, confirmation of this information may help to raise awareness of the problem amongst policy makers and the medical community. This could lead to future initiatives more specifically addressed in patients with chronic HF to minimise healthcare inequalities.

The strengths of this study include the publication of a protocol and search strategy ahead of beginning data extraction, which provides a reference standard for the intended study objectives and methodology. The search strategy has been informed with expert help, piloted and draws on validated SIGN search strategies. The methodology throughout is based on PRISMA guidance, which we are reporting against (Additional file 1). We anticipate including studies with large numbers of patients, followed up for many years with a clearly defined, discrete primary outcome, which we believe will allow us to provide precise summary measures. The observational, international focus of the included studies will make the findings generalisable to patients with chronic HF across healthcare settings.

Potential limitations include the limitation of associations that can be drawn from observational studies, which are not controlled for any factors which might influence outcomes. HF is a complex condition and classifications have changed across time, which may impact on the comparability of different study populations and make meta-analysis impossible due to the heterogeneity between studies.

We anticipate the results of this review being relevant to a range of stakeholders, including patient groups, the medical community, policy makers and researchers. To ensure we reach as wide an audience as possible, the results of this systematic review will be published in peer-reviewed international medical journals and presented both locally and nationally to relevant healthcare and patient groups. The review fits into the ongoing work by the Nuffield Department of Primary Care Health Sciences looking at the prognosis and management of HF in the community.

### Additional file

Additional file 1: PRISMA-P 2015 Checklist. (DOCX 32 kb)

## Appendix

**Table 1:** Search strategy for MEDLINE

Results from the search run on October 9, 2017		
1	heart failure.ti. or *heart failure/	88,434
2	cardiac failure.ti. or *heart failure/	83,752
3	congestive cardiac failure.mp. or *congestive heart failure/	84,241
4	1 or 2 or 3	89,628
5	mortality/ or cardiovascular mortality/ or mortality.mp.	622,213
6	death/ or death.mp.	662,318
7	survival.mp. or median survival time/ or long term survival/ or overall survival/ or survival/	1,009,482
8	outlook.mp.	9112
9	prognosis/ or prognosis.mp.	626,586
10	5 or 6 or 7 or 8 or 9	2,294,090
11	Clinical study/	3015
12	Longitudinal study/	119,557
13	Retrospective study/	705,410
14	Prospective study/	495,514
15	Randomized controlled trials/	121,449
16	Cohort analysis/	232,290
17	(Cohort adj (study or studies)).mp.	302,021
18	(follow up adj (study or studies)).tw.	43,831
19	(observational adj (study or studies)).tw.	67,469
20	(epidemiologic$ adj (study or studies)).tw.	71,816
21	survival analysis/	124,907
22	14 not 15	490,244
23	or/11-13,16-22	1,637,345
24	stable.mp.	409,429
25	chronic.mp.	1,132,332
26	community/ or community.mp.	452,486
27	primary care.mp. or primary medical care/	88,789
28	general practice.mp. or general practice/	42,899
29	family practice.mp. or general practice/	75,691
30	general population.mp. or population/	107,253
31	epidemiolog*.mp.	399,272
32	24 or 25 or 26 or 27 or 28 or 29 or 30 or 31	2,485,198
33	4 and 10 and 23 and 32	3282
34	limit 33 to (“all adult (19 plus years)” and humans)	2866

## Abbreviations

ACEI: Angiotensin-converting-enzyme inhibitors
BB: Beta-blockers
ESC: European Society of Cardiology
GRADE: Grading of Recommendations Assessment, Development and Evaluation working group
HF: Heart failure
MeSH: Medical subject headings
MRA: Mineralocorticoid receptor antagonists
NHS: National Health Service
PRISMA-P: Preferred Reporting Items for Systematic Reviews and Meta-Analysis Protocols
PROSPERO: International Prospective Register of Systematic Reviews
QUIPS: Quality in Prognosis Studies
SIGN: Scottish Intercollegiate Guideline Network
STROBE: Strengthening the Reporting of Observational Studies in Epidemiology
UK: United Kingdom
